# High-Throughput Immunogenetics Reveals a Lack of Physiological T Cell Clusters in Patients With Autoimmune Cytopenias

**DOI:** 10.3389/fimmu.2019.01897

**Published:** 2019-08-21

**Authors:** Donjete Simnica, Simon Schliffke, Christoph Schultheiß, Nicola Bonzanni, Lorenzo F. Fanchi, Nuray Akyüz, Barbara Gösch, Christian Casar, Benjamin Thiele, Janina Schlüter, Ansgar W. Lohse, Mascha Binder

**Affiliations:** ^1^Department of Internal Medicine IV, Oncology and Hematology, Martin-Luther-University Halle-Wittenberg, Halle, Germany; ^2^Department of Oncology and Hematology, BMT With Section Pneumology, University Cancer Center Hamburg, University Medical Center Hamburg-Eppendorf (UKE), Hamburg, Germany; ^3^ENPICOM B.V., ‘s-Hertogenbosch, Netherlands; ^4^Department of Gastroenterology With Sections Infectious and Tropical Diseases, University Medical Center Hamburg-Eppendorf (UKE), Hamburg, Germany; ^5^Bioinformatics Core, University Medical Center Hamburg-Eppendorf (UKE), Hamburg, Germany

**Keywords:** autoimmunity, AIC, CLL, GLIPH, immunosequencing, NGS, biomarker

## Abstract

Autoimmune cytopenias (AIC) such as immune thrombocytopenia or autoimmune hemolytic anemia are claimed to be essentially driven by a dysregulated immune system. Using next-generation immunosequencing we profiled 59 T and B cell repertoires (*TRB* and *IGH*) of 25 newly diagnosed patients with primary or secondary (lymphoma-associated) AIC to test the hypothesis if these patients present a disease-specific immunological signature that could reveal pathophysiological clues and eventually be exploited as blood-based biomarker. Global *TRB* and *IGH* repertoire metrics as well as *VJ* gene usage distribution showed uniform characteristics for all lymphoma patients (high clonality and preferential usage of specific *TRBV*- and *TRBJ* genes), but no AIC-specific signature. Since T cell immune reactions toward antigens are unique and polyclonal, we clustered TCRβ clones *in-silico* based on target recognition using the GLIPH (grouping of lymphocyte interactions by paratope hotspots) algorithm. This analysis revealed a considerable lack of physiological T cell clusters in patients with primary AIC. Interestingly, this signature did not discriminate between the different subentities of AIC and was also found in an independent cohort of 23 patients with active autoimmune hepatitis. Taken together, our data suggests that the identified T cell cluster signature could represent a blood biomarker of autoimmune conditions in general and should be functionally validated in future studies.

## Introduction

Autoimmune cytopenias (AIC) mainly comprise autoimmune hemolytic anemia (AIHA), immune thrombocytopenia (ITP), and autoimmune neutropenia (AIN). These disorders are driven by immune mediated destruction of mature hematopoietic cells in the periphery. Additionally, in rare cases AIC can be due to an immune mediated maturation defect in an otherwise normal bone marrow manifesting as pure red cell aplasia (PRCA) or acquired amegakaryocytic thrombocytopenia (AATP). The clinical course of AIC is highly diverse, ranging from subclinical presentation with abnormal laboratory findings to severe anemia, bleeding, or infection (1–3). While in some cases a clinical trigger cannot be identified, a considerable fraction of cases presents as secondary diseases in the context of an underlying malignancy (e.g., Non-Hodgkin's Lymphoma, NHL) or systemic autoimmune disease. Patients with chronic lymphocytic leukemia (CLL) have an especially high risk of developing AIC which is commonly attributed to their dysregulated immune system (4). The risk in CLL is reported to be higher in patients with adverse cytogenetics, unmutated *IGHV* of the malignant CLL clone and specific stereotyped B cell receptor configurations (5–10). Classical AIC cases rarely represent a diagnostic challenge for the hematologist, but the diagnosis may also be more intricate e.g., in cases showing overlapping patterns of immune reactivity with precursor and mature blood cells (11–16). When diagnosed, most cases of AIC—whether primary or secondary—are primarily treated with steroids (17). Second line treatments are much less defined, including other immunomodulatory drugs, growth factor receptor agonists, or splenectomy (18–20). Despite the plurality of therapeutic options, the rate of durable remissions is rather unsatisfactory to date (3). Moreover, reliable response predictive biomarkers are lacking.

Proposed mechanisms underlying AIC are: (i) Breakdown of central and/or peripheral tolerance resulting in T cell and autoantibody production against precursor and mature blood cells, (ii) complement-dependent cytotoxicity (CDC) of these autoantibodies causing blood cell clearance by macrophages in the reticuloendothelial system in spleen and liver, (iii) cellular cytotoxicity mediated by CD8+ T cells and natural killer cells (13, 21–23).

In order to gain new insights into the pathogenesis of AIC and identify signatures of autoreactive immune repertoires that could potentially be used in the diagnosis and/or follow-up of these patients, we used state-of-the-art immunosequencing technology. This let us dissect composition, dynamics and diversity of the T and B cell immune repertoire in a cohort of 25 patients with primary and secondary AIC with available follow-up biosamples. We found blood T cell signature in AIC patients consisting in a lack of physiological T cell clusters, which we confirmed in a different autoimmune cohort of 23 patients with active autoimmune hepatitis (AIH). Future studies will need to define the functional role of these clusters.

## Materials and Methods

### Study Approval

Informed consent was obtained from all patients and healthy donors (HD) for the use of their peripheral blood (PB) as approved by the ethics commission Hamburg (Ethikkommission der Ärztekammer Hamburg, Germany, project numbers PV4767 and PV4081). The study has been performed in accordance with the declaration of Helsinki of 1975.

### Patient Characteristics

Patient PB samples were collected between July 2012 and September 2018. The cohort comprised 25 patients with AIC. The diagnosis and response assessment was based on the criteria reported previously in the guidelines of the American Society of Hematology. In line with the distribution pattern of AIC, our cohort consisted essentially of patients with ITP (*n* = 12) and AIHA (*n* = 8), while only few patients had AIN, PRCA, or AATP (Table 1). Eleven of our patients had primary AIC, while 14 patients had secondary disease with an underlying lymphoid malignancy; the majority thereof was associated with CLL. As a reference and control cohorts, 43 PB samples of HD (Mean/Median Age: 56/59 years, 22 female), 14 samples from untreated patients with CLL without AIC (Mean/Median Age: 65/63 years, 5 female) and 23 PB samples of patients with active autoimmune hepatitis (AIH) were used (Table 1, Supplementary Table 1).

**Table 1:** Patient and sample characteristics.

**Patient**	**Age [y]**	**Diagnosis**	**Baseline**	**Follow-up at persistent disease**	**Follow-up at remission/inactive disease**
			**(no Tx)**	**Number of samples**	
**IMMUNE THROMBOCYTOPENIA (ITP)**					
ITP 1	21–25	ITP	1	0	0
ITP 2	86–90	CLL + ITP	1	0	0
ITP 3	71–75	CLL + ITP	1	3 on ibrutinib 2 on RB 1 w/o treatment	0
ITP 4	71–75	CLL + ITP	1	0	0
ITP 5	61–65	NHL^*^+ ITP	1	0	0
ITP 6	40–45	ITP	1	0	0
ITP 7	76–80	ITP	1	0	0
ITP 8	71–75	CLL + ITP	1	1 on steroids	0
ITP 9	61–65	ITP	1	0	1 on steroids
ITP 10	61–65	ITP	1	1 on steroids	0
ITP 11	46–50	ITP	1	1 on steroids	0
ITP 12	81–85	ITP	1	0	1 on steroids
**AUTOIMMUNE HEMOLYTIC ANEMIA (AIHA)**					
AIHA 1	61–65	AIHA	1	0	1 on steroids
AIHA 2	61–65	AIHA	1	1 on steroids	0
AIHA 3	61–65	CLL + AIHA	1	1 on ibrutinib	0
AIHA 4	81–85	CLL + AIHA	1	0	0
AIHA 5	56–60	CLL + AIHA	1	0	1 on RB
AIHA 6	76–80	CLL + AIHA	1	1 on steroids1 on rituximab	1 on venetoclax
AIHA 7	46–50	CLL + AIHA	1	0	0
AIHA 8	81–85	CLL + AIHA	1	0	0
**EVANS SYNDROME**					
AIHA/ITP	41–45	AIHA + ITP	1	1 on steroids	0
**PURE RED CELL APLASIA (PRCA)**					
PRCA 1	61–65	CLL + PRCA	1	0	7 on ibrutinib
**AUTOIMMUNE NEUTROPENIA (AIN)**					
AIN 1	71–75	CLL + AIN	1	0	5 on ibrutinib
**AQUIRED AMEGAKARYOTIC THROMBOCYTOPENIC PURPURA (AATP)**					
AATP 1	95–100	AATP	1	0	0
**AUTOIMMUNE HEPATITIS (AIH)**					
AIH 2	46–50	AIH + PBC	0	1 on Pred/Aza	0
AIH 3	56–60	AIH + PBC	0	1 on Azathioprin and Budesonid	0
AIH 4	46–50	AIH	0	1 on Pred/Aza	0
AIH 5	51–55	AIH	0	1 on Prednisolon and Infliximab	0
AIH 6	56–60	AIH	0	1 on Pred/Aza	0
AIH 7	31–35	AIH	0	1 on Prednisolon	0
AIH 9	20–25	AIH	0	1 on Pred/Aza	0
AIH 13	46–50	AIH + PSC	0	1 on Pred/Aza	0
AIH 14	66–70	AIH + PBC	1	0	0
AIH 16	21–25	AIH	1	0	0
AIH 18	45–50	AIH + PBC	0	1 on Pred/Aza	0
AIH 22	76–80	AIH	0	1 on Pred/Aza	0
AIH 25	31–35	AIH	0	1 on Pred/Aza	0
AIH 27	76–80	AIH	0	1 on Azathioprin	0
AIH 28	71–75	AIH	0	1 on Pred/Aza	0
AIH 29	31–35	AIH	0	1 on Pred/Aza	0
AIH 31	61–65	AIH	0	1 on Pred/Aza and Metformin	0
AIH 32	26–30	AIH	0	1 on Pred/Aza	0
AIH 35	71–75	AIH + PBC	0	1 on Pred/Aza	0
AIH 36	66–70	AIH + PBC	0	1 on Pred/Aza	0
AIH 37	61–65	AIH	1	0	0
AIH 40	36–40	AIH	0	1 on Prednisolon and Mercaptopurinin	
AIH 41	46–50	AIH+PBC	0	1 on Ursodeoxycholic acid (UDCA)	0

CLL, Chronic Lymphocytic Leukemia; NHL, Non-Hodgkin Lymphoma; PBC, Primary Biliary Cholangitis; Pred/Aza, Prednisolon/Azathioprin; PSC, Primary Sclerosing Cholangitis; RB, Rituximab/Bendamustine; Tx, Treatment;

* *low-grade Lymphoma, not otherwise specified*.

### Multiplex PCR for T Cell Receptor Beta (TRB) and Immunoglobulin Heavy Chain (IGH) Repertoire Amplification for Illumina Targeted Next-Generation Sequencing (NGS)

The *TRB* and *IGH* genes containing the entire *V, D*, and *J* gene segments were amplified with *TRB*-A and –B and *IGH* primer pools, respectively, from 250 ng PB genomic DNA, which corresponds to ~37,500 genome equivalents (24). Compared to our previously published protocol, the *TRB* PCR assay was refined by using a touch-down PCR protocol and a mixed primer *TRB*-A/B tube. In two consecutive PCR reactions amplicons were tagged with Illumina adapters and indices as previously described (25, 26). PCRs were performed using Phusion HS II (Thermo Fisher Scientific Inc., Germany). Amplicons were purified after agarose gel electrophoresis using the NucleoSpin^®^ Gel and PCR Clean-up kit (Macherey-Nagel, Germany). Before being subjected to NGS the concentration and quality of the amplicons/libraries was determined using Qubit (QIAGEN, Germany) and Agilent 2100 Bioanalyzer (Agilent technologies, Germany), respectively.

### Illumina Next-Generation Sequencing (NGS) and Data Analysis

NGS and de-multiplexing was performed on an Illumina MiSeq sequencer (600–cycle single indexed, paired-end run). Analysis of the *TRB* and *IGH* locus was computed using the MiXCR analysis tool versions 2.1.12 and 3.0.6, respectively (27, 28). At the alignment step the default MiXCR library was used for *TRB* sequences and the external IMGT library v3 (29) was used for *IGH* sequences. Each unique complementarity-determining region 3 (CDR3) nucleotide sequence was defined as one clone. Only productive sequences with a read count ≥2 were included in the analysis. Analyses were carried out and data plotting was performed using R (version 3.4.4) (30) and packages tidyverse (31), tcR (32), ade4 (33), as well as GraphPad Prism 7 (San Diego, CA). A *P*-value of < 0.05 was considered statistically significant.

### Calculation of Repertoire Metrics

We plotted diversity curves with alpha-modulated sensitivity [alpha-parameterized diversity (34)] to model scenarios where the relatively rare clones are weighed differentially as opposed to single diversity measures (see below) that have a defined sensitivity for clones in the repertoire. Diversity profiles of each cohort were calculated using the following equation:

Dα (f)=(∑i=1nfiα)11−a

**Figure 1:**
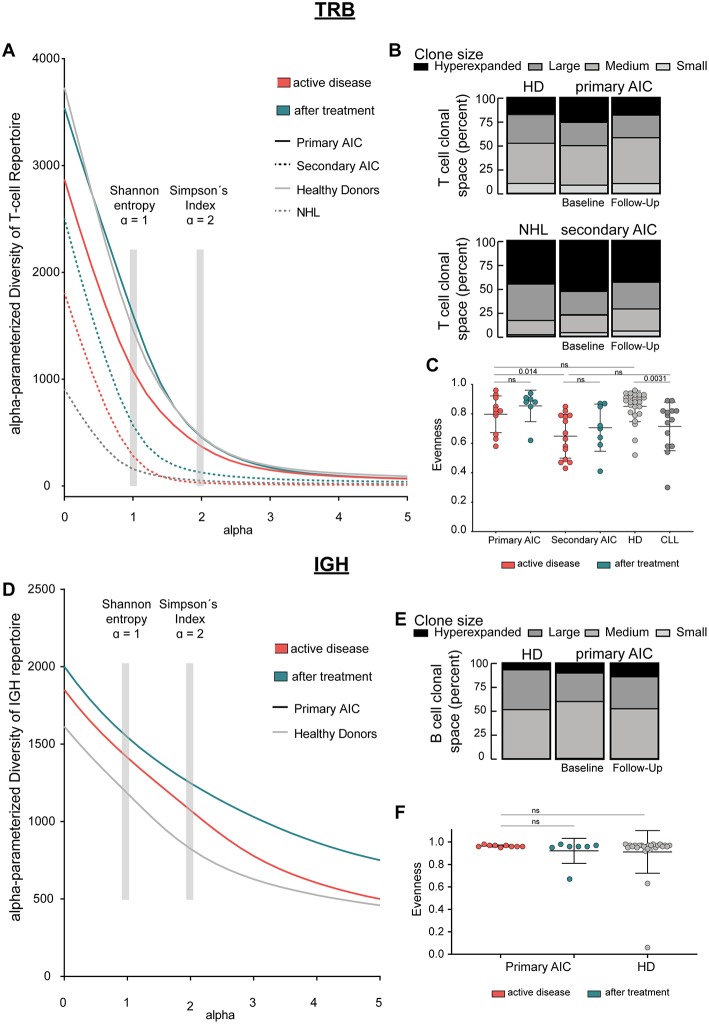
Immune repertoire metrics. **(A)** Alpha-parameterized diversity of *TRB* repertoire. The mean diversity profile curves of *TRB* repertoires at active disease and after treatment samples of patients with primary and secondary AIC, respectively, and HD and patients with CLL are shown. **(B)** Clonal T cell space of patients with primary and secondary AIC at active disease and after treatment, respectively, HD and patients with CLL. The distribution of small, medium, large, and hyperexpanded clones in % within the clonal T cell space is shown. **(C)** *TRB* Evenness (mean±standard deviation) of patients with primary and secondary AIC at active disease and after treatment, respectively, HD and patients with CLL. **(D)** Alpha-parameterized diversity of *IGH* repertoire. The mean diversity profile curves of *IGH* repertoires at active disease and after treatment samples of patients with primary AIC and HD are shown. **(E)** Clonal B cell space of patients with primary AIC at active disease and after treatment and HD. The distribution of small, medium, large, and hyperexpanded clones in % within the clonal B cell space is shown. **(F)** *IGH* Evenness (mean ± standard deviation) of patients with primary AIC at active disease and after treatment and HD. Clone sizes are defined as hyperexpanded (0.01 < x ≤ 1), large (0.001 < x ≤ 0.01), medium (1E-04 < x ≤ 0.001), small (1E-05 < x ≤ 1E-04). Gray boxes highlight alpha = 1, which resembles Shannon-Index, alpha =2, which resembles Simpson's Index. Statistical analysis: Two-sided unpaired *t*-test. Sample sizes in all panels are: Active disease primary AIC (*n* = 11), secondary AIC (*n* = 14); after treatment primary AIC (*n* = 7), secondary AIC (*n* = 8); HD (*n* = 25), and patients with CLL (*n* = 14).

As α increases, high frequency clones are weighed more. We generated diversity profile curves for α = 0 to α = 5, in steps of 0.2 using the R script kindly provided by Dr. V. Greiff of University of Oslo, Institute of Clinical Medicine. Two special cases of alpha are Shannon index (α = 1) and the Simpson's index (α = 2) – highlighted by gray bars in Figure 1.

The Shannon index (H) is a commonly used measure of diversity (18), which can be calculated as follows:

$$\begin{array}{l} H=\;\overset{S}{\underset{i=1}{\sum }}p_{i}\log_{2}p_{i}\; \end{array}$$

Where, S is the number of species/clones (richness) and p is the proportion of each clone within the repertoire. *p* = n/N, *n* = read count of each individual clone and *N* = the sum of all reads in the repertoire.

Evenness is calculated from H and Hmax (evenness = H/Hmax) with Hmax being the maximal possible value of H, if every clone in the repertoire was present at the same frequency.

The Simpson index (D) (35) is another measure used to quantify biological diversity in a given sample and is calculated as follow:

$$\begin{array}{l} D=\;\sum {\left(\frac{n}{N}\right)}^{2} \end{array}$$

where n is the read count of each individual clone and N = the sum of all reads in the repertoire.

### Calculation of Physio-Chemical Properties of *IGH* Data

The grand average of hydropathy index [GRAVY (36)] was calculated using BRepertoire (37). All GRAVY indices were ordered in ascending fashion for each cohort, respectively, and plotted against the cumulative relative frequency of each CDR3 amino acid sequence (1/total number of clones within cohort).

### PCA of Combinatorial VJ Usage

The combinatorial *VJ* usage was calculated using the gene.usage function of the tcR package (32). The dudi.pca function of the ade4 package (33) transformed the frequencies of each *VJ* combination (*n* = 650) within two cohorts tested to principal components to find linear association of different *VJ* combinations, that separate different individuals corresponding to different disease states.

### *In silico* GLIPH (Grouping of Lymphocyte Interactions by Paratope Hotspots)

We applied the GLIPH algorithm on our NGS generated AIC, HD, CLL, and AIH *TRB* dataset as described elsewhere (38). Briefly, we analyzed the following sets of NGS *TRB* data matched by age and read depth: primary AIC vs. HD, secondary AIC vs. CLL and active AIH vs. HD. Each cluster basically represents a consensus sequence of the CDR3 amino acid sequences it consists of. The majority of clusters found in each of the scenarios were shared between the respective cohorts analyzed. In order to compare the differences of “cluster size,” we calculated the Log_2_ fold-change of the mean CDR3 amino acid frequency. Log_2_ fold-change for clusters which were present exclusively in one cohort were set to artificial Log_2_ values for plotting purposes. We used the Wilcoxon-rank test to test if the mean frequencies were different between cohorts. A *P*-value of <0.05 was considered statistically significant.

## Results

### Immunosequencing of AIC Patients and Control Cohorts

We asked if patients with AIC showed a disease-specific immune signature of their PB T and B cells and if this potential signature was associated with response to immunomodulatory treatment. Therefore, we performed *TRB* and *IGH* NGS on 25 AIC patients with active disease and on 34 follow-up samples in the course of immunomodulatory treatment. As a control group, we used 25 age-matched HD for the primary AIC cohort and 14 age-matched patients with CLL for the secondary AIC cohort. We included an independent cohort of 23 patients with active AIH as control cohort for the GLIPH analysis. The median number of distinct *TRB* and *IGH* clonotypes per sample was 2,350 and 1,388, respectively. The samples were sequenced with a median sequencing depth of 51 575 reads per sample for *TRB* NGS (total of 7.7 million reads) and 26 915 for *IGH* NGS (total of 1.5 million reads), overview of sequencing results is shown in Supplementary Table 2.

### Broad Repertoire Metrics Analysis

Since clonal expansion and diversity represent important indicators for ongoing immune responses, we analyzed measures of T and B cell repertoire diversity and evenness in our AIC cases—before and on immunomodulatory treatment—as well as in age-matched healthy and CLL control cases. We generated diversity profile curves as described in the methods section to show the whole diversity spectrum of the repertoires. Generally, high diversity samples present higher y-values for each given alpha. We found that patients with primary AIC and healthy control cases had a more diverse PB *TRB* repertoire compared to patients with secondary AIC or the respective CLL control cases. This was reflected by a higher repertoire richness (at alpha = 0) as well as diversity profiles which overall ran at a higher level in primary AIC and healthy controls (Figure 1A). Moreover, the relatively balanced distribution of small, medium, large and hyperexpanded clones within the T cell clonal space of primary AIC and healthy controls confirmed the significantly more even repertoires in these individuals compared to secondary AIC and CLL control cases (Figures 1B,C). Overall, the *TRB* repertoire shifts mostly reflected a lymphoma bias and did not appear to be associated with autoimmunity (Figures 1A–C).

*IGH* repertoire analysis was only feasible for patients with primary AIC and healthy control cases since the repertoires of the secondary AIC and CLL cohorts were largely biased toward the malignant B cell clone. Mean PB *IGH* repertoire diversity of patients with primary AIC was numerically higher than in healthy control cases, however not significantly (Figure 1D). The B cell clonal space distribution and evenness as well as CDR3 length distribution and hydrophobicity properties of primary AIC were comparable to healthy controls (Figures 1E,F and Supplementary Figure 1).

### VJ Usage of Peripheral Blood T and B Cells in Patients With AIC

**Figure 2:**
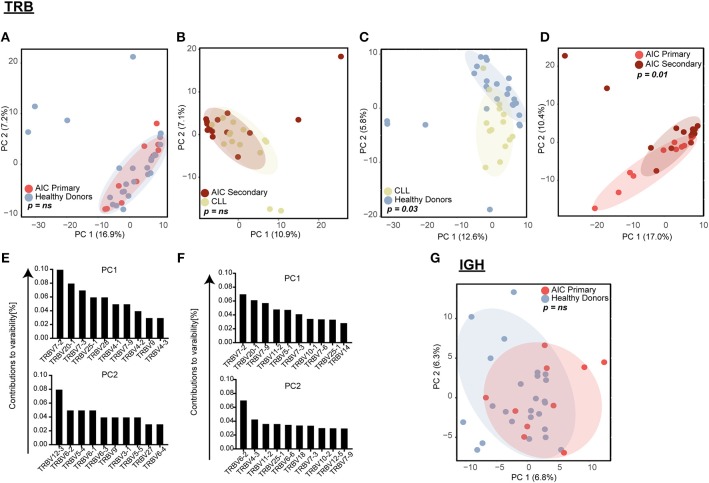
Principal component analysis (PCA) of *VJ* gene usage. *TRB VJ* PCA of **(A)** primary AIC vs. HD, **(B)** secondary AIC vs. CLL, **(C)** HD vs. CLL, and **(D)** primary vs. secondary AIC. Ten highest contributing variables for PCA of HD vs. CLL **(E)** and primary vs. secondary AIC **(F)** for each principal component in descending order are shown. **(G)** *IGH VJ* PCA for primary AIC vs. HD. Statistical analysis: Pillai–Bartlett test of MANOVA of principal components 1 and 2. AIC, Autoimmune Cytopenia; *IGHV*, Immunoglobulin heavy-chain V gene; CLL, Chronic Lymphocytic Leukemia; PC, Principal Component; *TRBV*, T cell receptor beta V gene.

While global repertoire metrics appeared to point rather toward lymphoma-biased repertoire differences between patients with primary or secondary AIC compared to respective control cohorts, we set out to screen all immune repertoires for the presence of individual expanded T and B cell receptor configurations present in patients with active AIC. Therefore, the combinatorial VJ gene usage of the T and B cell repertoire for patients with primary and secondary AIC and respective control groups were subjected to principal component analyses (Figure 2). Primary AIC cases clustered together with the healthy cohort, suggesting no specific *TRB VJ* usage signature (Figure 2A). TRB VJ gene combinations were clearly biased in patients with lymphoma vs. all other cohorts (Figures 2B–D). Some of the major biases in repertoire distributions of lymphoma patients consisted in a preferential usage of *TRBV7, TRBV6* and *TRBV12-3* genes (Figures 2E,F). Also *IGH VJ* usage did not reveal any specificities of primary AIC (Figure 2G). Secondary cases could not be analyzed due to the dominance of the malignant *IGH* clone in these repertoires.

### Clustering of T Cells With Common Antigen Recognition

**Figure 3:**
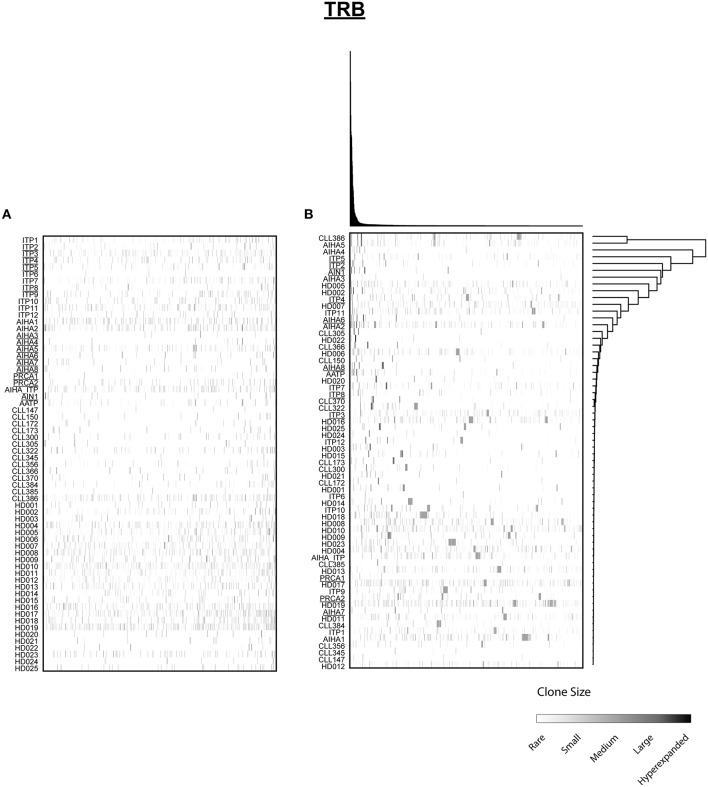
Distribution of shared T cell clones in AIC and control cohorts. T cell clones that were shared between at least 3 cases out of 64 patients and HD are shown. **(A)** Clustering by disease entity/control group. **(B)** Unsupervised hierarchical clustering. Color code illustrates frequency of individual clone within a repertoire. Secondary AIC Patients are underlined.

Next, we mined all repertoires for shared T cell receptor sequences characteristic for AIC in general or specific AIC subgroups. Out of the 64 patient or control cases, we identified a total number of 1066 CDR3 amino acid sequences shared between at least three individuals (Figure 3A). Many of these clones were shared by different cohorts and some of these represented known public clones that had been previously annotated in public databases (Sequences in Supplementary Table 3). As expected, unsupervised hierarchical clustering of the shared clonotypes did not reveal any biases (Figure 3B).

**Figure 4:**
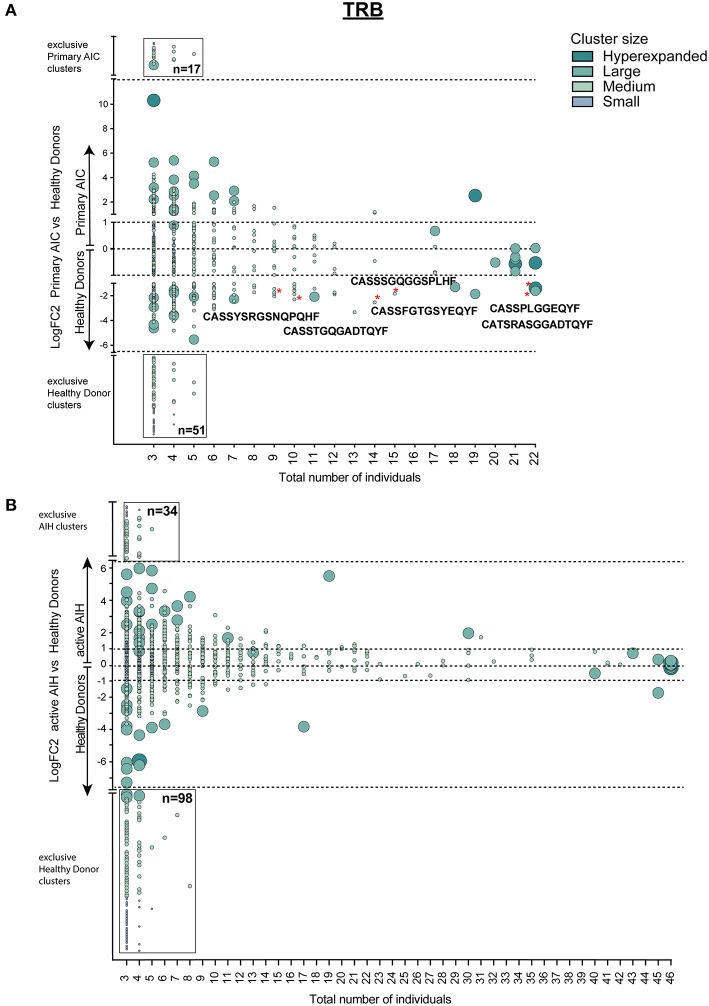
Cluster analysis of autoimmune *TRB* repertoires. Logarithmic fold-change of clusters that were present in at least 3 individuals in **(A)** patients with primary AIC vs. HD and **(B)** patients with active AIH vs. HD. Cluster size and color represents mean frequency of CDR3 clones that constitute the respective cluster. Cluster sizes are defined as hyperexpanded (0.01 < x ≤ 1), large (0.001 < x ≤ 0.01), medium (1E-04 < x ≤ 0.001), small (1E-05 < x ≤ 1E-04). Significantly different abundant cluster were marked with ^*^and their respective cluster sequence. Statistical test: Wilcoxon-rank test. ^*^p ≤ 0.05.

Since T cell immune reactions toward antigens are unique and polyclonal we clustered TCRβ clones based on target recognition using GLIPH (grouping of lymphocyte interactions by paratope hotspots) to identify disease-specific clusters involved in the pathogenesis of AIC. Primary AIC and HDs were analyzed together, and 657 clusters present in at least three individuals were found (Figure 4A). We identified clusters that were significantly overrepresented in HDs indicating that these were physiological *TRB* clusters (Figure 4A, marked with red asterisks). The numbers of clusters exclusively found in AIC or HD were 17 vs. 51, respectively, suggesting that patients with an autoimmune condition lack physiological T cell clusters compared to HDs (Figure 4A black-rimmed, Sequences in Supplementary Table 4).

To confirm our finding, we performed the T cell clustering analysis on an independent group of patients with active autoimmune hepatitis (AIH) using the T cell repertoires of 23 age- and sex-matched HDs as a reference. The analysis revealed 919 clusters that were present in at least 3 individuals (Figure 4B). Strikingly, and analogous to the analysis in primary AIC, HD presented almost a 3-fold higher number of exclusive *TRB* clusters than AIH (Figure 4B, black-rimmed, Sequences in Supplementary Table 4).

**Figure 5:**
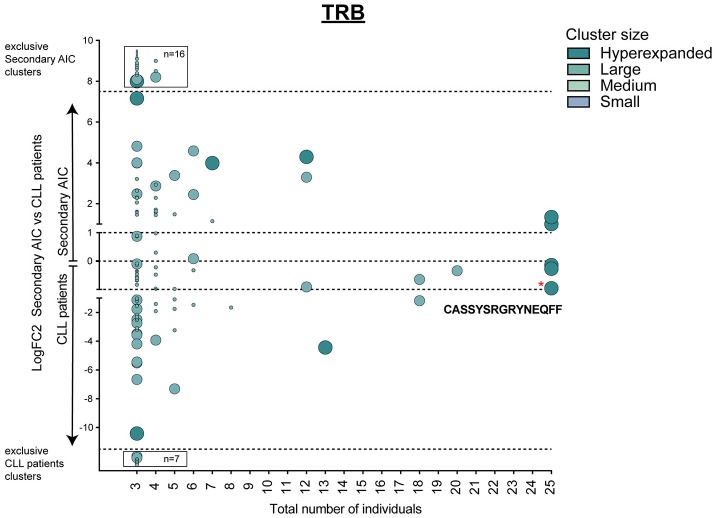
Cluster analysis of lymphoma-associated *TRB* repertoires. Logarithmic fold-change of clusters that were present in at least 3 individuals in patients with secondary AIC vs. patients with CLL. Cluster size and color represents mean frequency of CDR3 clones that constitute the respective cluster. Cluster sizes are defined as hyperexpanded (0.01 < x ≤ 1), large (0.001 < x ≤ 0.01), medium (1E-04 < x ≤ 0.001), small (1E-05 < x ≤ 1E-04). Significantly different abundant cluster were marked with ^*^and their respective cluster sequence. Statistical test: Wilcoxon-rank test. ^*^p ≤ 0.05.

This signature of “physiological *TRB* cluster lack” was not seen when comparing patients with secondary AIC to patients with CLL (Figure 5), indicating a high similarity of these repertoires that was likely driven by the underlying lymphoma.

## Discussion

Autoimmune cytopenias (AIC) are driven by dysregulated immune interactions resulting in the production of blood cell and/or precursor cell directed autoantibodies and T cells. Pathophysiologically these conditions are complex and it remains unclear to what extent certain immune abnormalities are causative in disease pathogenesis or epiphenomena in the inflammatory processes. Also, initiating and perpetuating factors in these diseases are insufficiently understood to date.

Immunosequencing has opened up new avenues for in-depth analysis of immune repertoires in autoimmune conditions (39). We used this technology to study peripheral blood T and B cell repertoires in a cohort of 25 patients with active AIC and in the course of treatment. We found that baseline *IGH* and *TRB* immune metrics such as diversity measures as well as *VJ* gene usage are not informative in discriminating cases with primary AIC from healthy controls or patients with secondary AIC from CLL patients (40, 41). Yet, in individual patients that responded to treatment, we observed increases in repertoire diversity and evenness reflecting the replacement of preexisting by novel small clones upon immunomodulation (28, 42). As expected, we found a clear lymphoma signature consisting in a highly contracted T cell repertoire and preferential *TRB VJ* gene usage in secondary AIC and CLL controls compatible with previous reports (43).

The clustering of our *TRB* data into groups of T cell clones with presumably identical antigen recognition revealed a significant lack or underrepresentation of several *TRB* clusters found physiologically in healthy individuals compared to cases with AIC. This suggests that the lack of certain T cell clusters—potentially of tolerogenic nature–could be involved in the pathogenesis of these autoimmune disorders. The functional properties of these T cell clusters, however, need to be confirmed in future experiments in order to shed further light on the pathophysiology of AIC or other autoimmune conditions. Interestingly, only patients with primary AIC could be differentiated from healthy controls by use of the clustering algorithm while the secondary cases were overall very similar to the lymphoma cohort. This suggested that the strong lymphoma-associated signature superimposed potential AIC- or autoimmunity-specific changes.

Since our immune signature of AIC was very consistent across the different AIC subentities, we reasoned that this immune configuration should be permissive for the development of immunologically-mediated cytopenias in general, without being lineage-specific. Also clinically, patients may develop combined ITP and AIHA (Evan's syndrome), suggesting common immunological grounds that may facilitate the development and persistence of autoreactive immune cell clones directed against one or more blood lineages or even blood precursor cells. Autoimmunity in general is assumed to arise when mechanisms conferring immune tolerance break down (44) and the paradox association of autoimmune disorders with immune deficiency and infections (45) implies underlying common factors or immune signatures, that contribute to alterations in immune response that could result in completely different autoimmune conditions. With this in mind, we tested if the lack of physiological T cell clusters holds true for other autoimmune conditions as well, where the immunological attack is directed against non-blood cell antigens. Intriguingly, we confirmed our findings in patients with AIH, an autoimmune condition with an often chronic T cell directed immune response against hepatic antigens. This finding corroborates the idea of an autoimmune specific T cell signature spanning disease entities.

Taken together, our data suggests an immunological signature of autoimmunity that may be derived from a simple blood test using next-generation immunosequencing. Our *in-silico* approach needs further corroboration especially to fully understand the functional role of the clusters. As a future perspective we propose that this signature should be prospectively studied in clinical trials.

## Data Availability

The datasets generated for this study can be found in the European Nucleotide Archive (ENA). http://www.ebi.ac.uk/ena/data/view/PRJEB33806.

## Author Contributions

MB, DS, and SS designed study and wrote manuscript. DS, SS, CS, BG, and NA performed DNA extraction and PCR amplification. NB and LF performed GLIPH analyses. DS and CC wrote R scripts for data analysis. MB, DS, and AL analyzed and interpreted the data. NA and BG optimized PCR amplification. JS collected and processed blood samples. AL and BT provided samples. All authors critically reviewed and approved the manuscript.

### Conflict of Interest Statement

NB and LF are employed by company ENPICOM B.V. The remaining authors declare that the research was conducted in the absence of any commercial or financial relationships that could be construed as a potential conflict of interest.

## Supplementary Material

Supplementary Figure 1 Physio-chemical properties of *IGH* repertoire in primary AIC and Healthys. Mean length distribution (+ standard deviation) **(A)** and Grand Average of hydropathy Index (GRAVY) of *IGH* CDR3 sequences in primary AIC with active disease and healthy controls. Statistical tests: Multiple *t*-tests **(A)** and Kolmogorov–Smirnov test **(B)**.

Supplementary Table 1 CLL clonotypes of secondary AIC patients and CLL control patients.

Supplementary Table 2 Overview of sequencing results.

Supplementary Table 3 Shared CDR3 amino acid sequences.

Supplementary Table 4 Exclusive TRB clusters of healthy donors.
