# Supplementary figures and images for: High-Throughput Immunogenetics Reveals a Lack of Physiological T Cell Clusters in Patients With Autoimmune Cytopenias
Source: Front Immunol. 2019 Aug 21;10:1897. doi: 10.3389/fimmu.2019.01897 (PMC6713037; doi:10.3389/fimmu.2019.01897)

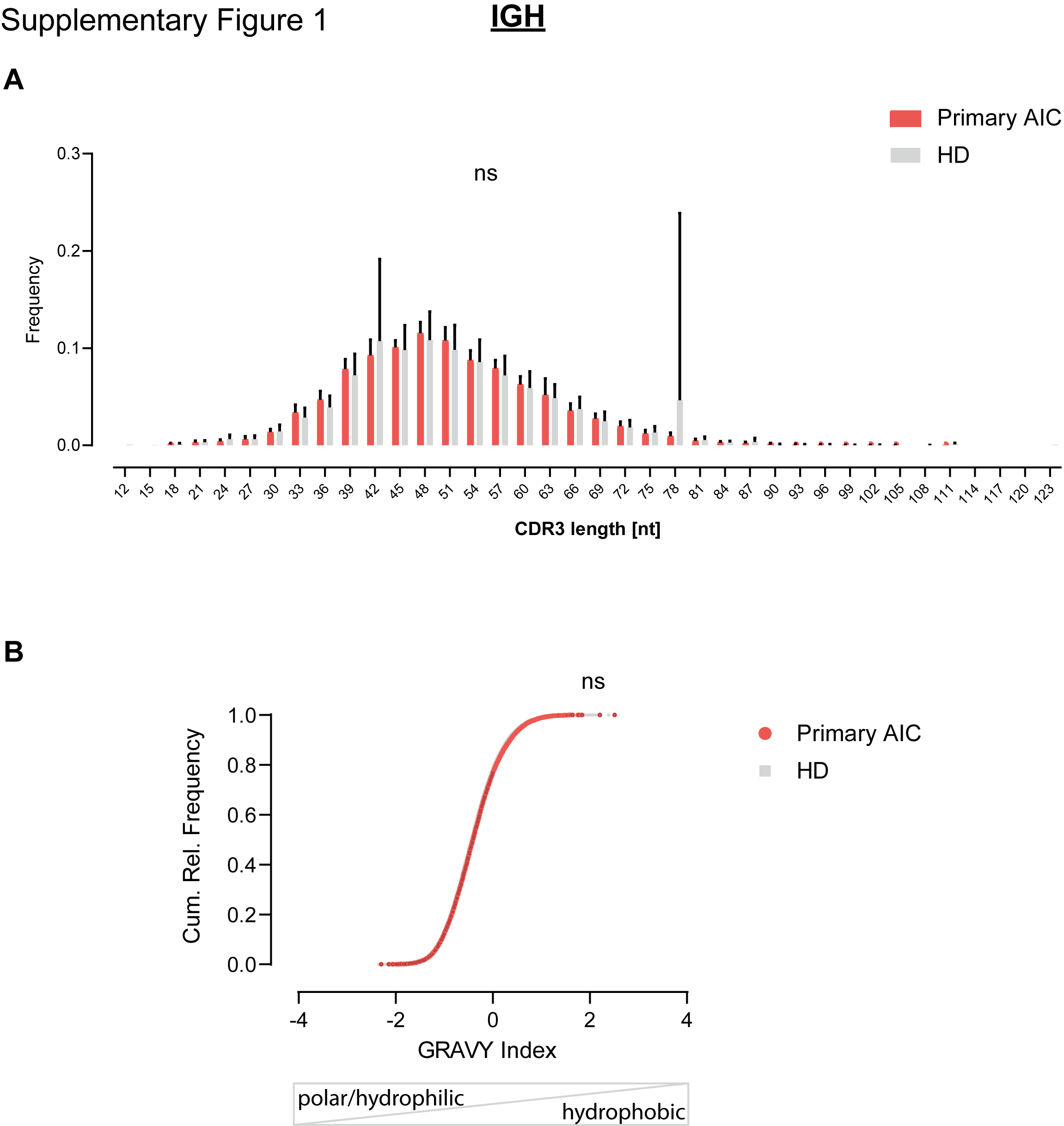

Supplement: Supplementary Figure 1 — Physio-chemical properties of IGH repertoire in primary AIC and Healthys. Mean length distribution (+ standard deviation) (A) and Grand Average of hydropathy Index (GRAVY) of IGH CDR3 sequences in primary AIC with active disease and healthy controls. Statistical tests: Multiple t-tests (A) and Kolmogorov–Smirnov test (B). [file Image_1.TIF]
