# Supplementary material for: High-Throughput Immunogenetics Reveals a Lack of Physiological T Cell Clusters in Patients With Autoimmune Cytopenias
Source: Front Immunol. 2019 Aug 21;10:1897. doi: 10.3389/fimmu.2019.01897 (PMC6713037; doi:10.3389/fimmu.2019.01897)
Supplement: Supplementary Table 1 — CLL clonotypes of secondary AIC patients and CLL control patients. [file Table_1.DOCX]

| Supplementary Table 1. CLL clonotypes of secondary AIC patients and CLL control patients. | | | | | | | | |
| --- | --- | --- | --- | --- | --- | --- | --- | --- |
| **Patient** | **IGHV** | **IGHD** | **IGHJ** | **CDR3 (AASeq)** | **Mutational status, %** | | **stereotype subset #1 or #7 ^1,2^** | **genetics (FISH Del17p, Del11q)** |
| Secondary AIC patients | | | | | | | | |
| ITP 2 | 1-18 | 5-12 | 6 | CARVPVPYSGYEDLGYYYYMDVW | 98.18 | UM | no | none |
| ITP 3 | 6-1 | 7-27 | 4 | CATDLTGDHFDSW | 96.57 | M | no | n.e. |
| ITP 4 | 3-23 | 6-19 | 4 | CAKDDQQLGLPEDYW | 90.99 | M | no | none |
| ITP 5 | 3-7 | 3-22 | 4 | CARDSSGYRPLDYW | 97.65 | M | no | n.e. |
| ITP 8 | 3-7 | 3-10 | 6 | CARDPGPLLWFRELLSTYYYYGMDVW | 100 | UM | no | Del11q |
| AIHA 3 | 1-46 | 3-22 | 4 | CARDLYYYDSSGYYSGFFDYW | 99.55 | UM | 12 | Del11q |
| AIHA 4 | n.e. | | | | | | | Del17p |
| AIHA 5 | 4-34 | 3-10 | 4 | CARGSGSGSYFTPFDSW | 90.37 | M | 11 | Del17p |
| AIHA 6 | n.e. | | | | | | | Del17p |
| AIHA 7 | 1-18 | 6-19 | 4 | CARVGWYSEYYFDYW | 100 | UM | 1 | n.e. |
| AIHA 8 | 3-7 | 3-3 | 6 | CARGGTTYYDFWSGPFYYYYGMDVW | 100 | UM | no | n.e. |
| PRCA 1 | 3-30 | 6-19 | 5 | CARVGVHSSGWYVDVRTTLGPWFDPW | 98.18 | UM | no | none |
| PRCA 2 | 4-55 | 3-3 | 6 | CARGESYYDFWSGYPSVLGYYGMDVW | 94.52 | M | 7 | none |
| AIN 1 | 3-71 | 5-12 | 4 | CAKDVSAGRLGVAAAFDYW | 91.08 | M | no | none |
| CLL Patients (w/o AIC) | | | | | | | | |
| CLL 147, m, 62y | 1-69 | 2-2 | 6 | CARESPDIVVVPAAIDYYYGMDVW | 99.55 | UM | no | Del11q |
| CLL 150, m, 67y | 4-39 | 3-16 | 3 | CAYVLGAFDMW | 93.58 | M | no | none |
| CLL 172, m, 59y | n.e. | | | | | | | |
| CLL 173, m, 55y | 3-23 | 6-6 | 4 | CAKAQRDYSSSSVDYW | 92.27 | M | no | none |
| CLL 300, m, 64y | 3-64 | 2-15 | 4 | CVKKSGYCHGGTCYYEYW | 90.19 | M | no | Del17p |
| CLL 305, f, 69y | 4-59 | 4-17 | 4 | CVRRGKGDYDDYGTFFDLW | 91.66 | M | no | none |
| CLL 322, m, 59y | 3-11 | 3-9 | 6 | CARDTNERYFDWLLDAPYYMDVW | 100 | UM | no | none |
| CLL 345, f, 76y | 2-5 | 3-3 | 4 | CAHRRAAPLYYDFWSGYYYYFDYW | 100 | UM | no | none |
| CLL 356, m, 68y | 3-21 | 5-12 | 6 | CARDGGYSGYDLFHYYYYYMDVW | 99.09 | UM | no | Del17p |
| CLL 366, f, 82y | 3-30 | 4-17 | 5 | CARGGHGDYAFAFDPW | 98 | UM | no | none |
| CLL 370, m, 62y | 3-11 | 3-9 | 6 | CARDTNERYFDWLLDAPYYMDVW | 100 | UM | no | Del11q |
| CLL 384, f, 49y | 1-69 | 2-8 | 6 | CARVRGPYCTNGVCYISAGLVDYYYYMDVW | 99.54 | UM | no | none |
| CLL 385, f, 76 | 1-69 | 4-17 | 5 | CALASSPAAVTNLGWFDPW | 93.48 | M | no | none |
| CLL 386, m, 61y | 4-34 | 3-10 | 4 | CARGSGSGSYFTPFDSW | 90.37 | M | no | Del17p |

AASeq – Amino Acid sequence, f – female, CDR3 – Heavy chain complementarity determining region 3, m – male, M – mutated, n.e. not evaluated, UM – unmutated, y- years

1. Stamatopoulos K, Belessi C, Moreno C, et al. Over 20% of patients with chronic lymphocytic leukemia carry stereotyped receptors: Pathogenetic implications and clinical correlations. Blood. 2007;109(1):259-270.

2. Agathangelidis A, Darzentas N, Hadzidimitriou A, et al. Stereotyped B-cell receptors in one-third of chronic lymphocytic leukemia: a molecular classification with implications for targeted therapies. Blood. 2012;119(19):4467-4475.
